# The role of beta-arrestin2 in shaping fMRI BOLD responses to dopaminergic stimulation

**DOI:** 10.1007/s00213-017-4609-6

**Published:** 2017-04-05

**Authors:** Kristoffer Sahlholm, Giovanna D. Ielacqua, Jinbin Xu, Lynne A. Jones, Felix Schlegel, Robert H. Mach, Markus Rudin, Aileen Schroeter

**Affiliations:** 1 Institute for Biomedical Engineering, University and ETH Zurich, Wolfgang-Pauli-Str. 27, 8093 Zurich, Switzerland; 2 Department of Radiology, Mallinckrodt Institute of Radiology, Washington University School of Medicine, 510 S. Kingshighway Blvd, St. Louis, MO 63110 USA; 3 Department of Neuroscience, Karolinska Institutet, Retzius väg 8, SE-171 77 Stockholm, Sweden; 4 Department of Radiology, Perelman School of Medicine, University of Pennsylvania, 231 S. 34th St, Philadelphia, PA 19104 USA; 5 Neuroscience Center Zurich, University and ETH Zurich, Winterthurer-Str. 190, 8057 Zurich, Switzerland; 6 Institute of Pharmacology and Toxicology, University of Zurich, Winterthurer-Str. 190, 8057 Zurich, Switzerland

**Keywords:** Arrestin3, Dopamine, Dopamine receptor, Dopamine agents, Eticlopride

## Abstract

**Rationale:**

The dopamine D_2_ receptor (D_2_R) couples to inhibitory G_i/o_ proteins and is targeted by antipsychotic and antiparkinsonian drugs. Beta-arrestin2 binds to the intracellular regions of the agonist-occupied D_2_R to terminate G protein activation and promote internalization, but also to initiate downstream signaling cascades which have been implicated in psychosis. Functional magnetic resonance imaging (fMRI) has proven valuable for measuring dopamine receptor-mediated changes in neuronal activity, and might enable beta-arrestin2 function to be studied in vivo.

**Objectives:**

The present study examined fMRI blood oxygenation level dependent (BOLD) signal changes elicited by a dopamine agonist in wild-type (WT) and beta-arrestin2 knockout (KO) mice, to investigate whether genetic deletion of beta-arrestin2 prolongs or otherwise modifies D_2_R-dependent responses.

**Methods:**

fMRI BOLD data were acquired on a 9.4 T system. During scans, animals received 0.2 mg/kg apomorphine, i.v. In a subset of experiments, animals were pretreated with 2 mg/kg of the D_2_R antagonist, eticlopride.

**Results:**

Following apomorphine administration, BOLD signal decreases were observed in caudate/putamen of WT and KO animals. The time course of response decay in caudate/putamen was significantly slower in KO vs. WT animals. In cingulate cortex, an initial BOLD signal decrease was followed by a positive response component in WT but not in KO animals. Eticlopride pretreatment significantly reduced apomorphine-induced BOLD signal changes.

**Conclusions:**

The prolonged striatal response decay rates in KO animals might reflect impaired D_2_R desensitization, consistent with the known function of beta-arrestin2. Furthermore, the apomorphine-induced positive response component in cingulate cortex may depend on beta-arrestin2 signaling downstream of D_2_R.

**Electronic supplementary material:**

The online version of this article (doi:10.1007/s00213-017-4609-6) contains supplementary material, which is available to authorized users.

## Introduction

Dopamine signaling is crucially involved in a multitude of physiological brain functions, such as motor program selection, goal-directed behavior, and endocrine homeostasis. Dopamine receptors, in particular, the dopamine D_2_ receptor (D_2_R) constitute important drug targets for treatment of neuropsychiatric, neurological, and neuroendocrine disorders, such as Parkinson’s disease, schizophrenia, and hyperprolactinemia (Beaulieu and Gainetdinov 2011). Dopamine receptors, which belong to the super-family of G-protein coupled receptors (GPCRs), are known to signal via several downstream pathways, which include the classical G protein-pathway, and the more recently described arrestin pathway. The main postsynaptic effector mechanism of dopamine in the striatum has long been considered to be the modulation of adenylate cyclase via the coupling of D_2_-like (D_2_R, D_3_R, D_4_R) and D_1_-like (D_1_R, D_5_R) receptors to inhibitory G_i/o_- and stimulatory G_s_ proteins, respectively.

However, the important role of the arrestin pathway in dopaminergic signaling has become increasingly apparent during the past decade. Arrestins are a family of proteins known to bind GPCRs following their activation by agonist, thus blocking further receptor-G protein interactions and initiating GPCR internalization (Claing et al 2002). Whereas, the role of arrestins in receptor desensitization and internalization is well-established, recent studies have demonstrated that arrestins can also scaffold other signaling proteins, including protein phosphatase 2A (PP2A) and Akt, a protein kinase which has been genetically linked to psychosis risk (Emamian et al. 2004). Beta-arrestin2 (also known as arrestin3) has been specifically implicated as the arrestin isoform regulating D_2_R internalization as well as downstream signaling cascades (Beaulieu et al. 2005; Peterson et al. 2015; Skinbjerg et al. 2009). Notably, beta-arrestin2 knock out (KO) mice show a reduced locomotor response to the direct-acting dopamine agonist, apomorphine, and the dopamine-releasing agent, amphetamine (Beaulieu et al. 2005).

All clinically used antipsychotics antagonize beta-arrestin2 recruitment to the D_2_R, suggesting that this mechanism might be relevant for their therapeutic activity (Klewe et al. 2008; Masri et al. 2008). It has been proposed that the clinical benefits of antipsychotics are mediated mainly via inhibition (or in the case of aripiprazole, partial agonism) of the beta-arrestin2 pathway, whereas attenuation of G protein activation would be responsible for extrapyramidal side effects. Accordingly, efforts are underway to develop D_2_R ligands which specifically modulate arrestin signaling (Allen et al. 2011; Park et al. 2016). Novel data from the Caron lab (Urs et al. 2016) suggest that ligands such as UNC9994 (Allen et al. 2011), which are partial agonists at the arrestin pathway, exert antipsychotic-like actions in rodent models of schizophrenia by activating the D_2_R-arrestin pathway preferentially in cortical, as compared to striatal regions, due to the higher expression of beta-arrestin2 in cortex. Taken together, the present evidence suggests that the arrestin pathway is an important aspect of dopaminergic signaling and that dysregulation of this pathway might play a key role in states of altered dopamine signaling, such as schizophrenia (Freyberg et al. 2010). A measure of beta-arrestin2 activity which could be obtained in vivo might thus be useful in translational studies.

Functional magnetic resonance imaging (fMRI) is an attractive, non-invasive method for measuring neuronal activity in the living brain, and has proven valuable for investigating dopaminergic signaling in a variety of contexts, including cognitive and psychiatric studies in human subjects and investigations of receptor function as well as drug development in experimental animals (see, e.g., Booij and van Amelsvoort 2012; Bruns et al. 2015; Cools and D'Esposito 2011; Knutson and Gibbs 2007; Mandeville et al. 2014). Beta-arrestin2 KO mice have previously been used to characterize the contribution of beta-arrestin2 to dopamine-dependent behaviors, as well as to study the effects of D_2_R internalization on PET radioligand binding following amphetamine administration (Beaulieu et al. 2005; Skinbjerg et al. 2010). However, the role of beta-arrestin2 in brain hemodynamic responses to dopaminergic challenges has not yet been explored. Hence, the present study was designed to examine blood oxygenation level dependent (BOLD) fMRI responses to the dopamine agonist, apomorphine, in caudate/putamen (CPu) and cingulate cortex (Cg) of wild-type (WT) and beta-arrestin2 KO mice.

## Materials and methods

### Animals

Male beta-arrestin2 KO mice (Bohn et al. 1999) and age-matched C57BL/6 J WT counterparts were purchased from the Jackson Laboratory (Bar Harbor, ME), and were between 3 and 6 months of age when used in experiments.

### Drugs

*R*-(−)-Apomorphine hydrochloride was purchased from Sigma-Aldrich (St. Louis, MO), *S*-(−)-eticlopride from Abcam chemicals (Cambridge, UK), and UNC9994 was custom-synthesized by Axon Medchem B.V. (Groningen, the Netherlands). Eticlopride was dissolved in saline, apomorphine in saline supplemented with 1 mM ascorbic acid (Merck, Darmstadt, Germany; pH 7.4 with NaOH), and UNC9994 was dissolved in saline supplemented with 15% *w*/*v* (2-hydroxypropyl)-β-cyclodextrin (Sigma-Aldrich).

### Autoradiography

Mice were sacrificed at 6 months of age, and their brains were immediately removed and fresh-frozen on powdered dry ice. Brains were sectioned at 20 μm on a cryostat and thaw-mounted onto Fisher Superfrost Plus slides (Thermo Fisher Scientific, Waltham, MA), with 12 sets of 12 sections per slide taken from the rostral through caudal striatum, corresponding approximately to Bregma +2.0 to −1.0 mm (Paxinos and Franklin 2001). Slides were stored at −80 °C until processing for autoradiography. [^3^H]SCH23390 (85 Ci/mmol), [^3^H]raclopride (76 Ci/mmol), and [^3^H]WIN35428 (76 Ci/mmol) were purchased from Perkin Elmer Life Sciences (Boston, MA). [^3^H]dihydrotetrabenazine ([^3^H]DTBZ; 20 Ci/mmol) was purchased from American Radiolabeled Chemicals (St. Louis, MO).

All sections were pre-incubated for 20 min in binding buffer (50 mM Tris, pH 7.4, 25 °C, containing 120 mM NaCl, 5 mM KCl) to remove endogenous receptor ligands. Following incubation with the respective radioligand in an open staining jar, slides were then rinsed five times at 1 min intervals with ice-cold buffer, and subsequently air dried and made conductive by coating the free side with a copper foil tape. The radioligand concentrations used were [^3^H]SCH23390, 3.5 nM; [^3^H]raclopride, 2.9 nM; [^3^H]WIN35428, 2.2 nM; [^3^H]DTBZ, 5.8 nM. Slides were then placed in a gas chamber containing a mixture of argon and triethylamine (Sigma-Aldrich) as part of a gaseous detector apparatus; the Beta Imager 2000Z Digital Beta Imaging System (Biospace Lab, Nesles la Vallée, France). After the gas was well mixed and a homogenous state was reached, further exposure for 12 h yielded high-quality images. A [^3^H]Microscale (American Radiolabeled Chemicals) was counted simultaneously as a reference for quantitative radioactivity analysis.

Data analysis was performed using the program Beta-Vision Plus (Biospace Lab). Using neuroanatomical landmarks (Paxinos and Franklin 2001), bilateral regions of interest (ROIs) were drawn freehand along the border of the entire striatum of serial sections from each individual mouse brain to define the representative striatal binding densities. Data were linearly fitted to a standard slope which was used for calibration, thereby converting counts per minute per mm^2^ into nCi per mg tissue. Subsequently, the radioligand binding densities were calculated using the specific activity of each radioligand as previously described (Xu et al. 2010). Nonspecific binding was determined by incubating slides with radioligand in the presence of an excess concentration of a cold competitor; 1 μM (+)-butaclamol ([^3^H]SCH23390), 1 μM *S*-(−)-eticlopride ([^3^H]Raclopride), 1 μM nomifensine ([^3^H]Win35428), and 1 μM *S*-(−)-tetrabenazine ([^3^H]DTBZ), respectively.

### Animal preparation for fMRI experiments

Animals were anesthetized with isoflurane (4% for induction, 2% for endotracheal intubation and during set-up on the animal cradle) in a 20% O_2_/80% air mixture. For reproducible positioning, the head of the animals was fixed using stereotactic ear bars. Ophthalmic ointment was applied to the eyes. A rectal temperature probe served to keep the animal at 36.0 ± 0.5 °C by means of a warm-water circuit as part of the animal cradle (Bruker Biospin GmbH, Ettlingen, Germany). After intubation and positioning on the cradle, mice were maintained under 1.5% isoflurane and artificial ventilation using a small animal ventilator (MRI-1; CWE Inc., Ardmore, PA), with a 20% O_2_/80% air mixture at a rate of 80 breaths/min, with a respiration cycle of 25% inhalation, 75% exhalation, and an inspiration volume of 1.8 ml/min. Pharmaceutical drugs were delivered via a cannula inserted into the tail vein (i.v.), or the nape of the animal (s.c.). 0.7 mg/kg pancuronium bromide (a neuromuscular blocking agent; Sigma-Aldrich), was administered i.v. before starting the fMRI experiments. In a subset of experiments, animals received 2 mg/kg eticlopride s.c. before starting fMRI data acquisition and 25 min prior to apomorphine administration.

### fMRI

Eleven WT and 15 beta-arrestin2 KO mice were imaged on a 9.4 T Bruker BioSpec 94/30 MR system (Bruker BioSpin) using a four-element cryogenic phased array surface receiver coil together with a linearly polarized room temperature volume resonator for transmission. Sixteen contiguous 0.5-mm thick slices were acquired with the most rostral slice at Bregma +3.5, with reference to a stereotaxic mouse brain atlas (Paxinos and Franklin 2001). An anatomical reference scan was obtained using a T_2_-weighted rapid acquisition with relaxation enhancement (RARE) sequence: field of view (FOV) = 23.66 × 14 mm^2^, matrix dimension (MD) = 338 × 200, resulting in an in-plane voxel dimension of 70 × 70 μm, repetition time (TR) = 3500 ms, echo time (TE) = 13 ms, effective echo time (TE_eff_) = 52 ms, RARE factor = 8, number of averages (NA) = 2. BOLD fMRI data was acquired with 1-s time resolution using a gradient-echo echo-planar imaging (GE-EPI) sequence: FOV = 16 × 7 mm^2^, MD = 80 × 35, yielding an in-plane voxel dimension of 200 × 200 μm, TR = 1000 ms, TE = 12 ms, flip angle (FA) = 60°, NA = 1. Before starting the fMRI sequence, local field homogeneity had been improved in the area of interest using previously acquired field maps. Each BOLD fMRI scan session lasted 40 min (2400 repetitions), out of which 10 min of baseline were recorded before drug administration. During scans, drugs were administered using an infusion pump (Harvard Apparatus, Holliston, MA) connected to an i.v. (apomorphine, UNC9994) or s.c. (eticlopride) cannula.

### fMRI data analysis

The first 300 volumes (i.e., 300 s of the 600 s (10 min) lasting baseline) of each data set were discarded to account of the T_1_ relaxation and for equilibration purposes. Drifts were removed from the BOLD signal using linear regression with the Statistical Parametric Mapping (SPM; http://www.fil.ion.ucl.ac.uk/spm/) function spm_filter (cut-off at 2100 repetitions = whole time series, thus corresponds to a linear detrend) and converted into percent relative to baseline.

As the concentration-time-curve for apomorphine and hence the response-model-function per defined brain voxel/region is unknown, a model-free visualization approach was chosen. Color-coded maps were generated with AFNI (http://afni.nimh.nih.gov/) to visualize the voxel-wise integral of the BOLD signal curve over the intervals 300 s (time point of apomorphine injection) to 600 s, 600 to 900 s, and 600 to 2100 s.

Time courses of BOLD signal changes were analyzed using the freeware plugin, Aedes (aedes.uef.fi), running under MATLAB (The MathWorks, Natick, MA), to draw ROIs for CPu and Cg with guidance of a stereotaxic mouse brain atlas (Paxinos and Franklin 2001). For visualization, time courses of BOLD signal changes extracted from the drawn ROIs were smoothed using a 5-s moving average. Fitting of monoexponential functions to time course data, in order to quantify response decay rates, was done in Matlab. Graph plotting was done in MS Excel (Microsoft, Redmond, WA), Origin (OriginLab, Northampton, MA), and GraphPad Prism (GraphPad Software, Inc., La Jolla, CA). Statistical analyses (Two-way ANOVA with Bonferroni-corrected pairwise comparisons, two-way repeated measures ANOVA, or Student’s *t*-test) were performed on BOLD signal data, to test the effects of genotype and treatment, and interactions between them, on the BOLD response to apomorphine in the respective ROIs, as described in the results.

### Measurement of systemic physiological parameters

To test the extent of possible effects that the administered dose of apomorphine exerts on systemic physiological variables, heart rate (beats per minute; bpm), O_2_ saturation (in %), and pulse distention (in μm) were measured using a pulse oximeter (MouseOx; Starr Life Sciences Corp., Oakmont, PA), with the optical sensor attached to the shaved flank of the mouse. The animal was anesthetized, intubated, and ventilated, and apomorphine was administered i.v. in the same way as during the fMRI scan protocol.

## Results

### Autoradiography

**Fig. 1:**
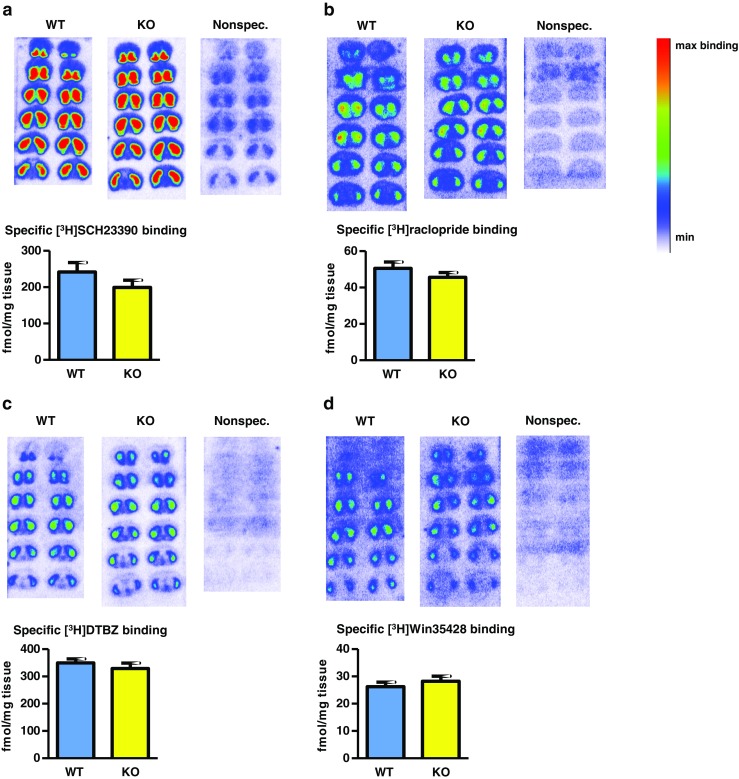
Quantitative autoradiographic comparison of dopamine marker densities in striata from WT and beta-arrestin2 KO mice. Representative autoradiograms of coronal sections through striata from WT and KO mice, incubated with the respective radioligands, are shown together with nonspecific binding autoradiograms, and the mean specific binding in striatum ( ± SEM) for each radioligand in the two genotypes. Nonspecific binding was determined by co-incubation of radioligand with excess concentrations of the appropriate competing, cold ligands, as described in Materials and methods. Binding densities were investigated for **a** the D_1_R radioligand; [^3^H]SCH232390, **b** the D_2/3_R radioligand; [^3^H]raclopride, **c** the VMAT2 radioligand; [^3^H]Win35428, and **d** the DAT radioligand; [^3^H]DTBZ. WT; *n* = 5, KO; *n* = 7–8. No statistically significant differences were detected (*p* > 0.05, Student’s *t*-test)

Quantitative autoradiography performed on coronal tissue sections through striata from WT and beta-arrestin2 KO mice using tritiated ligands for D_1_R, D_2/3_R, dopamine transporter (DAT), and vesicular monoamine transporter-2 (VMAT2) revealed no significant differences in average binding densities between the genotypes for all four radioligands (Fig. 1).

### fMRI

**Fig. 2:**
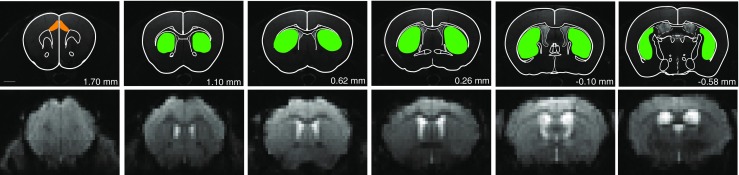
T_2_-weighted anatomical reference images showing superimposed ROIs for the brain regions analyzed; *green*, caudate/putamen; *orange*, cingulate cortex

**Fig. 3:**
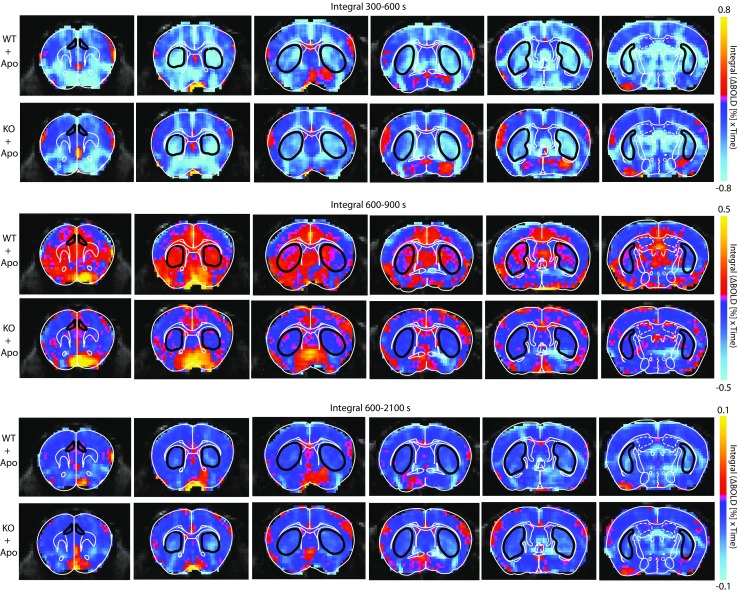
Color-coded maps to visualize voxel-wise integral of the BOLD signal curve over the intervals 300 s (time point of apomorphine injection) to 600 s, 600 to 900 s, and 600 to 2100 s post-apomorphine. *Upper panel*: For the interval 300 to 600 s, i.e., up to 5 min post-apomorphine, no obvious differences between BOLD signal changes for WT and KO were detected in CPu and Cg. *Middle panel*: For the interval of 600 to 900 s, i.e., 5 to 10 min post-apomorphine, a prolonged negative response component, i.e., a slower decay of the response in the KO CPu is represented by *blue* clusters, in contrast to *red clusters* for WT animals, in which the BOLD signal change negative response component appeared shorter. *Lower panel*: For the interval of 600 to 2100 s, i.e., 5 to 30 min post-apomorphine, *red-colored* regions reflect increased and positive integral values in WT Cg compared to KO Cg; however, no difference between WT and KO was detected in the CPu for that interval. The *color bar* indicates the BOLD signal change integral over the respective time period

**Fig. 4:**
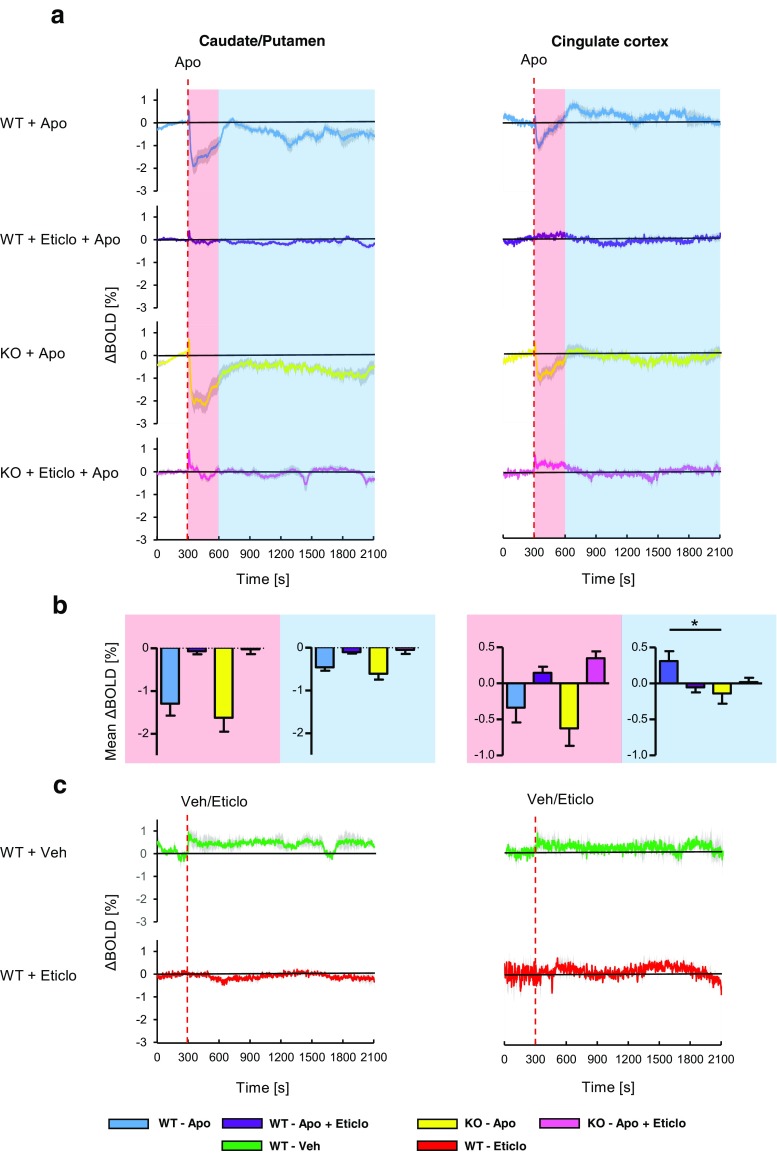
BOLD fMRI time course data. **a** BOLD signal changes in caudate/putamen (CPu) and cingulate cortex (Cg) in response to i.v. challenge with 0.2 mg/kg apomorphine (Apo) in WT and beta-arrestin2 KO animals, with or without pretreatment with 2 mg/kg eticlopride (Eticlo), s.c.; WT + Apo, *n* = 11; KO + Apo, *n* = 15; WT + Eticlo + Apo, *n* = 11; KO + Eticlo + Apo, *n* = 11. **b** Mean BOLD signal change in the respective brain regions during the time intervals indicated by shading in (**a**); *light red*, 0–5 min after Apo injection; *light blue*, 5–30 min after Apo injection. A two-way ANOVA revealed a significant main effect of eticlopride pretreatment, but not of genotype, nor any interaction between genotype and drug treatment, during 0–5 and 5–30 min in CPu and during 0–5 min in Cg, whereas during 5–30 min in Cg, there were no significant main effects but a significant interaction between genotype and eticlopride treatment. Bonferroni-corrected pairwise comparisons detected significant differences between WT and KO mice treated with apomorphine alone during 5–30 min in Cg, but not during 0–5 min in Cg, nor during either interval in CPu; *p < 0.05. **c** Control traces showing BOLD signal changes upon i.v. administration of vehicle (Veh; saline + ascorbic acid; *n* = 2) and s.c. administration of eticlopride (*n* = 2). Brain regions correspond to those in (**a**). All data are given as mean ± SEM. *Red dashed lines* indicate the time point of Apo, Veh, or Eticlo injection, respectively

For fMRI data analysis, ROIs were drawn for CPu and Cg (Fig. 2). Following i.v. administration of 0.2 mg/kg apomorphine, BOLD signal decreases were observed in CPu and Cg of WT and KO animals (Fig. 3 upper panel; Fig. 4a). During the first 5 min post-apomorphine, the mean BOLD change was −1.29 ± 0.28% (WT) and −1.62 ± 0.32% (KO) in CPu, and −0.34 ± 0.21% (WT) and −0.62 ± 0.24 (KO) in Cg (Fig. 4b). No obvious differences were detected between the genotypes neither for CPu nor for Cg, which is also reflected by the color-coded maps for BOLD signal change integrals of 300 to 600 s (i.e., up to 5 min post-apomorphine, Fig. 3 upper panel). After reaching a negative peak, the BOLD signal amplitude returned towards baseline. The mean BOLD signal change (relative to baseline) between 5 and 30 min after apomorphine injection was −0.46 ± 0.08% (WT) and −0.61 ± 0.14% (KO) in CPu (Fig. 4b). In Cg, the initial BOLD signal decrease during the first 5 min was followed by a positive response component in WT but not in KO animals (Fig. 4a). Between 5 and 30 min post-apomorphine, the mean BOLD signal change in Cg was 0.31 ± 0.14% (WT) and −0.14 ± 0.14% (KO), respectively (Fig. 4b). Red-colored regions on color-coded maps reflect increased and positive integral values in WT Cg compared to KO Cg for intervals of 600 to 2100 s (i.e., 5 to 30 min post-apomorphine), while as noted above, BOLD signal changes in CPu were similar between WT and KO during that interval (Fig. 3 lower panel). Pretreatment with eticlopride virtually abolished apomorphine-induced BOLD signal changes in both CPu and Cg (Fig. 4a).

There was a significant main effect of eticlopride pretreatment, but not of genotype, nor any interaction between genotype and drug treatment, during the first 5 and the last 25 min in CPu and during the first 5 min in Cg, (Two-way ANOVA; *F*_(1, 44)_ = 31.59, *p* < 0.001; *F*_(1, 44)_ = 18.12, *p* < 0.001; *F*_(1, 44)_ = 14.43, *p* < 0.001, for the main effect of eticlopride pretreatment in CPu during first 5 and last 25 min, and in Cg during the first 5 min, respectively). During the last 25 min in Cg, there was a significant interaction between the effects of genotype and drug treatment (Two-way ANOVA; *F*_(1, 44)_ = 4.83, *p* = 0.033), but no significant main effects of genotype or drug treatment. Pairwise comparisons revealed a significant difference between the effects of apomorphine, when administered alone, in WT compared to KO mice in Cg during the last 25 min (*p* < 0.05, with Bonferroni correction; Fig. 4b), but not during the first 5 min, nor during either interval in CPu. We also performed two-way repeated measures ANOVAs with time and genotype as factors, comparing the entire 35-min BOLD time courses with 1-min sampling for WT and KO mice receiving apomorphine alone, and found a significant effect of genotype in Cg (*F*_(1, 816)_ = 4.48, *p* = 0.045), but not in CPu (*F*_(1, 816)_ = 0.56, *p* = 0.462).

I.v. administration of vehicle (saline + ascorbic acid or saline + [2-hydroxypropyl]-β-cyclodextrin), or s.c. administration of 2 mg/kg eticlopride produced minor deviations of the BOLD signal from baseline (Fig. 4c; Supplementary Fig. 1). Vehicle induced positive changes in CPu and Cg, whereas eticlopride induced a slight BOLD signal decrease in CPu, and an increase in Cg. The beta-arrestin2-biased D_2_R ligand, UNC9994, was also tested in KO and WT mice. UNC9994 elicited BOLD signal increases in CPu and Cg in WT and KO animals at i.v. doses of 0.2, 0.07, and 0.007 mg/kg (Supplementary Fig. 1).

**Fig. 5:**
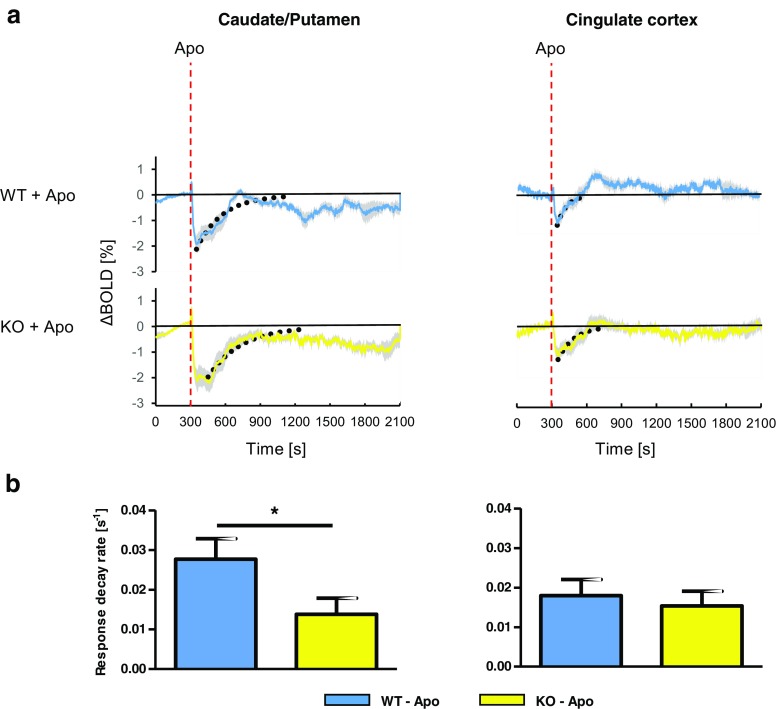
Comparison of response decay rates in caudate/putamen (CPu) and cingulate cortex (Cg) between WT and beta-arrestin2 KO mice. **a** Traces from WT and KO mice (same data as in Fig. 4a) showing BOLD signal changes upon administration of apomorphine (Apo; time point indicated by *red dashed lines*). *Dotted black curves* indicate fits of exponential functions to the data. **b** Estimated response decay rates. Exponential functions were fitted to individual BOLD signal traces, as exemplified by the fits to mean data in (**a**), to estimate rates of decay of the response to i.v. apomorphine. Traces were excluded from the analysis in cases where an exponential function could not be fitted. CPu WT, *n* = 9; CPu KO, *n* = 13; Cg WT, *n* = 8; Cg KO, *n* = 11; *p < 0.05; Student’s *t*-test. All data are shown as mean ± SEM

The time course of decay of the initial BOLD signal decrease following apomorphine was estimated by fitting an exponential function to individual traces, starting from the negative peak of the response (*y* = *A* × e^[−λ×*t*]^, where *y* is the BOLD signal amplitude at time *t*, A is the BOLD signal amplitude at the start of the fit, λ is the exponential decay constant, and *t* is time in seconds). The response decay rate was significantly slower in CPu of KO animals, as compared to WT (WT; 0.028 ± 0.005 s^−1^, KO; 0.014 ± 0.004 s^−1^, *p* = 0.045, Student’s *t*-test; Fig. 5), whereas in Cg, decay rates were similar between genotypes (WT; 0.018 ± 0.004 s^−1^, KO; 0.015 ± 0.004, *p* = 0.650; Fig. 5). On color-coded maps for intervals of 600 to 900 s (i.e., 5 to 10 min post-apomorphine; covering much of the decay time course in CPu) the prolonged negative response component, i.e., a slower decay of the response in the KO CPu, is represented by blue clusters, in contrast to red clusters for WT animals, in which the negative BOLD response component appeared shorter (Fig. 3 middle panel). While there was no significant difference in response decay time course between genotypes in Cg, WT animals showed more positive changes in this region compared to KO during the 600–900 s interval (Fig. 3 middle panel), reflecting the positive response component in Cg of WT animals, as noted above.

### Systemic cardiovascular effects of apomorphine treatment

**Fig. 6:**
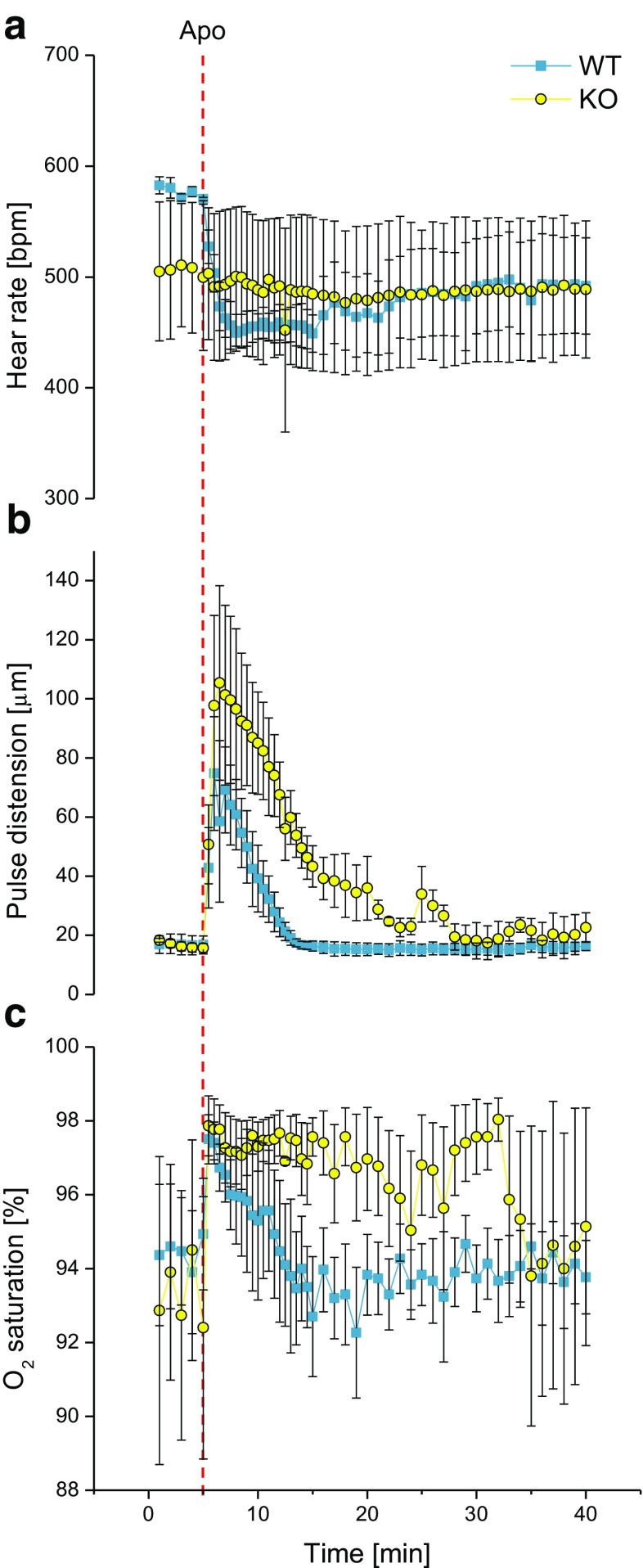
Monitoring of systemic physiological parameters during injection of apomorphine (0.2 mg/kg i.v.) in beta-arrestin2 KO and WT mice (*n* = 3 per genotype). Heart rate (**a**) (in beats per minute, bpm), pulse distention (**b**) (in μm), and O_2_ saturation (**c**) (in %) have been recorded in bench-top experiments carried out mimicking the conditions during the fMRI experiments. *Red dashed line* indicates the time point of apomorphine (Apo) injection. Data are shown as mean ± SEM

In bench-top experiments performed on animals undergoing the same protocol of anesthesia and artificial ventilation as during the fMRI scans, upon i.v. injection of 0.2 mg/kg apomorphine, heart rate decreased transiently from 576 ± 3 bpm in WT to 506 ± 61 bpm in KO mice during 5 min baseline prior to drug injection, to 469 ± 15 bpm in WT and 495 ± 64 bpm in KO mice during the first 5 min following drug injection. During the same intervals, pulse distention displayed increases from an average of 16.9 ± 3.2 μm in WT and 16.7 ± 1.3 μm in KO mice, to 55.7 ± 13.3 μm in WT and 90.7 ± 23.8 μm in KO mice. O_2_ saturation increased from 94.5 ± 1.6% in WT and 93.3 ± 3.3% in KO to 96.3 ± 1.2% in WT and 97.4 ± 0.8% in KO (Fig. 6); the values returned to baseline earlier in WT mice. None of the values were significantly different between genotypes (not shown).

## Discussion

Whereas previous studies have used beta-arrestin2 KO mice to investigate dopaminergic functions, the putative effect of the KO on the densities of dopaminergic markers has not been investigated with the precision provided by quantitative autoradiography. Using this approach, the present study found D_1_R, D_2_/_3_R, DAT, and VMAT2 densities to be similar between WT and beta-arrestin2 KO mice, suggesting that the KO does not alter the expression levels of other components of the dopamine system. In agreement with our [^3^H]raclopride data, Skinbjerg et al. (2010) reported similar binding of the D_2_/_3_R PET radiotracers, [^18^F] fallypride and [^11^C] methoxy-norpropylapomorphine, between WT and beta-arrestin2 KO mice.

Beta-arrestin2 has been implicated mainly downstream of D_2_R, rather than D_1_R, activation (Beaulieu et al. 2005; Peterson et al. 2015). However, in the present study, we chose to use the non-selective dopamine agonist, apomorphine, since this ligand was used in behavioral experiments with beta-arrestin2 KO mice, where the KO animals displayed significantly reduced locomotor activity as compared to WT (Beaulieu et al. 2005). Pretreatment of mice with the D_2_R antagonist, eticlopride, which has been previously used to probe the involvement of D_2/3_R signaling in fMRI responses evoked by pharmacological (Chen et al. 2005) and nociceptive stimulation (Shih et al. 2009, 2012; Chen et al. 2013), prevented apomorphine-induced BOLD signal changes in the studied brain regions. This suggests that D_2_R activation was responsible for the apomorphine-induced BOLD signal changes, which would be consistent with the low dose of apomorphine used in the present study, which was selected to minimize cardiovascular side effects, along with the 5–10-fold higher affinity of apomorphine for D_2_R vs. D_1_R (Millan et al. 2002).

The observed BOLD signal responses to apomorphine were negative in CPu, whereas in Cg, a smaller BOLD signal decrease was followed by a positive response component in WT, but not in KO animals. Both positive and negative response components were abolished by eticlopride pretreatment. Contrasting to the present findings, Ireland et al. (2005) found striatal BOLD signal increases in response to D_2_R agonist administration in rat, and Nguyen et al. (2000) and Schwarz et al. (2006), studying the effects of 2 mg/kg and 0.2 mg/kg apomorphine, respectively, on rat cerebral blood volume (CBV) changes, reported signal increases in striatum. Delfino et al. (2007) reported a positive BOLD signal change in rat striatum in response to 1.25 mg/kg apomorphine, as well as to a D_1_R-specific agonist, whereas no significant response was observed after administration of a D_2_R-selective agonist. However, several studies have reported negative striatal CBV changes in response to D_2_R activation in rat (Chen et al. 2005; Choi et al. 2006; Shih et al. 2009), whereas D_1_R agonism increased- and D_1_R antagonism decreased CBV in the same brain region (Choi et al. 2006). Similarly, Dixon et al. (2005) reported that positive BOLD responses resulting from administration of the dopamine releasing agent, amphetamine, were blocked by a D_1_R antagonist, whereas a D_2_R antagonist blocked BOLD signal changes in brain regions which showed negative responses. Hence, the weight of evidence suggests that D_1_R and D_2_R activation elicits hemodynamic responses which are mainly opposite in sign (Mandeville et al. 2013). The positive striatal BOLD and CBV responses observed with apomorphine in previous reports may thus reflect greater activation of D_1_R, relative to D_2_R, than was achieved in the present study.

Indeed, whereas D_1_R and D_2_R are present at similar densities in mouse, D1R density is relatively greater in rat, which might explain discrepancies between species, and also why dopamine-releasing agents such as amphetamine produce positive BOLD and CBV responses in rat striatum (Mandeville et al. 2013). Similarly, recent fMRI studies employing optogenetics to stimulate midbrain dopamine neuron activity reported CBV and BOLD signal increases in rat striatum (Decot et al. 2016; Ferenczi et al. 2016; Lohani et al. 2016).

Interestingly, recent data (Urs et al. 2016) indicate that arrestin-biased D_2_R ligands are able to recruit beta-arrestin2 only in cortical, as opposed to striatal, brain regions, presumably due to the higher expression of beta-arrestin2 in cortex, and that activation of the arrestin pathway has excitatory effects, in contrast to G protein-dependent effects downstream of D_2_R, which are in general inhibitory. Although inference about neuronal excitation vs. inhibition cannot be made on basis of the sign of BOLD signal changes (Lauritzen et al. 2012; Shih et al. 2009), the observations of Urs et al. (2016) seem to agree with the present results, which suggest that the positive component of the BOLD response to apomorphine in Cg is beta-arrestin2-dependent and elicited via D_2_R stimulation. In an attempt to corroborate the origin of this positive response component in Cg, we evaluated BOLD signal responses to the D_2_R ligand, UNC9994, which is a partial agonist at beta-arrestin2 recruitment to the D_2_R, while being unable to elicit G protein-dependent signaling through this receptor (Allen et al. 2011). However, because BOLD signal responses to UNC9994 appeared to be independent of beta-arrestin2, we did not further investigate the actions of this ligand in the present study. These arrestin-independent responses might reflect non-dopaminergic activity of UNC9994; indeed, this ligand has been reported to be an agonist at 5HT_1A_ and 5HT_2C_ receptors in vitro (Allen et al. 2011).

In CPu, but not in Cg, the rate of response decay (return of the BOLD signal towards baseline) was slightly but significantly slower in KO compared to WT animals. The slower rate of response decay in beta-arrestin2 KO mice would be in agreement with the known role of beta-arrestin2 in terminating D_2_R signaling through G proteins, and in mediating D_2_R internalization (Skinbjerg et al. 2009, 2010). However, the difference in decay rates between KO and WT animals was modest, suggesting a substantial contribution of additional factors besides beta-arrestin2 to response termination. For example, the pharmacokinetics of apomorphine in mouse brain might also play an important role in shaping the response decay rate. To address the impact of pharmacokinetics on response time course, fMRI measurements would need to be performed in parallel, or ideally, simultaneously, with PET imaging using D_2/3_R–specific radiotracers. Applying this approach to beta-arrestin2 KO and WT mice, as recently suggested by Sander et al. (2016), would enable the assessment not only of the contribution of D_2_R occupancy, but also that of receptor internalization, to the rate of decay of the apomorphine-induced BOLD signal response. The ongoing development of small animal imaging systems combining these two modalities (Wehrl et al. 2014) is expected to make this option more widely accessible in the near future.

The increases in O_2_ saturation and pulse distention and decreases in heart rate observed in response to apomorphine administration indicate that this drug exerts systemic cardiovascular effects also at the low dose employed in the present study. The changes in O_2_ saturation, and in particular, pulse distention, tended to be greater and longer lasting in KO mice, although the differences vs. WT were not significant. Beta-arrestin2 is expressed also in the cardiovascular system (Lymperopoulos and Bathgate 2013), and the trend towards greater and longer lasting systemic cardiovascular responses to apomorphine in KO mice may thus be related to diminished desensitization of the receptors mediating this response.

Blood pressure changes, which may override cerebral blood flow autoregulation, can influence the BOLD signal considerably (Gozzi et al. 2007; Wang et al. 2006). Kalisch et al. (2005) reported evidence that in rats, a higher dose of apomorphine (1 mg/kg, i.v.) elicited peripheral hypotensive effects which correlated with striatal brain BOLD signal decreases. It is thus important to monitor systemic hemodynamic parameters for effects of drug administration, in order to assess whether any such changes may confound the fMRI readout. However, it appears less likely that the BOLD signal changes observed in response to apomorphine in the present study are caused solely by such systemic effects, as the dominating BOLD signal changes are opposite in sign (negative) to what would be expected for an increase in pulse distention, (a measure which correlates with blood pressure; Olivera et al. 2010) and an increase in peripheral O_2_ saturation. Nevertheless, given the vasoactive actions of dopamine receptor agonists, which may occur on systemic as well as on local tissue level (Kalisch et al. 2005; Choi et al. 2006), a contribution of such direct vascular drug effects to the measured BOLD signal changes cannot be excluded in the present study, nor in previous fMRI studies of dopaminergic function (which often used higher doses of agonist).

Finally, the anesthetic used can interfere with fMRI responses. Several previous studies of the dopamine system used halothane (e.g., Chen et al. 2005; Choi et al. 2006; Dixon et al. 2005; Ireland et al. 2005; Schwarz et al. 2006) whereas Chen et al. (2013) and Shih et al. (2009); 2012) used alpha-chloralose, and Delfino et al. (2007) and Kalisch et al. (2005) used isoflurane. The volatile anesthetics, halothane and isoflurane, are known to induce prominent vasodilation which may alter cerebrovascular response characteristics, whereas alpha-chloralose has been suggested to interfere with the dopamine system in particular (Haensel et al. 2015; Mandeville et al. 2014). Thus, confounding effects of anesthesia are not possible to exclude in the present investigation, nor in previous studies in the field where anesthetics were used. However, when studying drug-induced fMRI responses, the use of anesthesia is often preferable to imaging of awake animals, which presents its own set of problems including the increased risk for motion-induced artifacts and changes in physiological parameters such as breathing rate (Mandeville et al. 2014), especially when administering dopaminergics and other drugs which might be expected to stimulate motor activity and autonomic functions.

In conclusion, the present study found prolonged D_2_R-dependent BOLD response decay rates in CPu in beta-arrestin2 KO animals, which might reflect impaired D_2_R desensitization, consistent with the known role of beta-arrestin2 in terminating G protein-dependent signaling by this receptor. Furthermore, a positive BOLD response component, apparently dependent on beta-arrestin2 signaling downstream D_2_R, was observed in Cg, but not in CPu. This response component may represent the cortex-specific excitatory effects recently reported for the D_2_R-arrestin pathway. The present results may be informative for further live imaging studies addressing signaling and desensitization mediated via beta-arrestin2, e.g., in animal models of psychiatric disease.

## Electronic supplementary material

Below is the link to the electronic supplementary material.

Supplementary Fig. 1 BOLD signal responses in WT and beta-arrestin2 KO mice to UNC9994 (UNC), administered i.v. at doses of 0.2 (WT, *n* = 5; KO, *n* = 6), 0.07 (WT, *n* = 2; KO, *n* = 2), and 0.007 mg/kg (WT, *n* = 3; KO, *n* = 2), and to vehicle (Veh; WT, *n* = 2), in caudate/putamen and in cingulate cortex. Red dashed lines indicate the time of injection of UNC or vehicle. All data are shown as mean ± SEM (GIF 209 kb)

High Resolution Image (TIFF 161 kb)

## References

[CR1] Allen JA, Yost JM, Setola V, Chen X, Sassano MF, Chen M, Peterson S, Yadav PN, Huang XP, Feng B, Jensen NH, Che X, Bai X, Frye SV, Wetsel WC, Caron MG, Javitch JA, Roth BL, Jin J (2011). Discovery of β-arrestin-biased dopamine D2 ligands for probing signal transduction pathways essential for antipsychotic efficacy. Proc Natl Acad Sci U S A.

[CR2] Beaulieu JM, Gainetdinov RR (2011). The physiology, signaling, and pharmacology of dopamine receptors. Pharmacol Rev.

[CR3] Beaulieu JM, Sotnikova TD, Marion S, Lefkowitz RJ, Gainetdinov RR, Caron MG (2005). An Akt/beta-arrestin 2/PP2A signaling complex mediates dopaminergic neurotransmission and behavior. Cell.

[CR4] Bohn LM, Lefkowitz RJ, Gainetdinov RR, Peppel K, Caron MG, Lin FT (1999). Enhanced morphine analgesia in mice lacking beta-arrestin 2. Science.

[CR5] Booij J, van Amelsvoort T, Rosenthal W (2012). Imaging as tool to investigate psychoses and antipsychotics. Handb Exp Pharmacol.

[CR6] Bruns A, Mueggler T, Künnecke B, Risterucci C, Prinssen EP, Wettstein JG, von Kienlin M (2015). “Domain gauges”: a reference system for multivariate profiling of brain fMRI activation patterns induced by psychoactive drugs in rats. NeuroImage.

[CR7] Chen YC, Choi JK, Andersen SL, Rosen BR, Jenkins BG (2005). Mapping dopamine D2/D3 receptor function using pharmacological magnetic resonance imaging. Psychopharmacology.

[CR8] Chen CC, Shih YY, Chang C (2013). Dopaminergic imaging of nonmotor manifestations in a rat model of Parkinson’s disease by fMRI. Neurobiol Dis.

[CR9] Choi JK, Chen YI, Hamel E, Jenkins BG (2006). Brain hemodynamic changes mediated by dopamine receptors: role of the cerebral microvasculature in dopamine-mediated neurovascular coupling. NeuroImage.

[CR10] Claing A, Laporte SA, Caron MG, Lefkowitz RJ (2002). Endocytosis of G protein-coupled receptors: roles of G protein-coupled receptor kinases and beta-arrestin proteins. Prog Neurobiol.

[CR11] Cools R, D'Esposito M (2011). Inverted-U-shaped dopamine actions on human working memory and cognitive control. Biol Psychiatry.

[CR12] Decot HK, Namboodiri VM, Gao W, McHenry JA, Jennings JH, Lee SH, Kantak PA, Jill Kao YC, Das M, Witten IB, Deisseroth K, Shih YI, Stuber GD (2016). Coordination of brain-wide activity dynamics by dopaminergic neurons. Neuropsychopharmacology.

[CR13] Delfino M, Kalisch R, Czisch M, Larramendy C, Ricatti J, Taravini IR, Trenkwalder C, Murer MG, Auer DP, Gershanik OS (2007). Mapping the effects of three dopamine agonists with different dyskinetogenic potential and receptor selectivity using pharmacological functional magnetic resonance imaging. Neuropsychopharmacology.

[CR14] Dixon AL, Prior M, Morris PM, Shah YB, Joseph MH, Young AM (2005). Dopamine antagonist modulation of amphetamine response as detected using pharmacological MRI. Neuropharmacology.

[CR15] Emamian ES, Hall D, Birnbaum MJ, Karayiorgou M, Gogos JA (2004). Convergent evidence for impaired AKT1-GSK3beta signaling in schizophrenia. Nat Genet.

[CR16] Ferenczi EA, Zalocusky KA, Liston C, Grosenick L, Warden MR, Amatya D, Katovich K, Mehta H, Patenaude B, Ramakrishnan C, Kalanithi P, Etkin A, Knutson B, Glover GH, Deisseroth K (2016). Prefrontal cortical regulation of brainwide circuit dynamics and reward-related behavior. Science.

[CR17] Freyberg Z, Ferrando SJ, Javitch JA (2010). Roles of the Akt/GSK-3 and Wnt signaling pathways in schizophrenia and antipsychotic drug action. Am J Psychiatry.

[CR18] Gozzi A, Ceolin L, Schwarz A, Reese T, Bertani S, Crestan V, Bifone A (2007). A multimodality investigation of cerebral hemodynamics and autoregulation in pharmacological MRI. Magn Reson Imaging.

[CR19] Haensel JX, Spain A, Martin C (2015). A systematic review of physiological methods in rodent pharmacological MRI studies. Psychopharmacology.

[CR20] Ireland MD, Lowe AS, Reavill C, James MF, Leslie RA, Williams SC (2005). Mapping the effects of the selective dopamine D2/D3 receptor agonist quinelorane using pharmacological magnetic resonance imaging. Neuroscience.

[CR21] Kalisch R, Delfino M, Murer MG, Auer DP (2005). The phenylephrine blood pressure clamp in pharmacologic magnetic resonance imaging: reduction of systemic confounds and improved detectability of drug-induced BOLD signal changes. Psychopharmacology.

[CR22] Klewe IV, Nielsen SM, Tarpø L, Urizar E, Dipace C, Javitch JA, Gether U, Egebjerg J, Christensen KV (2008). Recruitment of beta-arrestin2 to the dopamine D2 receptor: insights into anti-psychotic and anti-parkinsonian drug receptor signaling. Neuropharmacology.

[CR23] Knutson B, Gibbs SE (2007). Linking nucleus accumbens dopamine and blood oxygenation. Psychopharmacology.

[CR24] Lauritzen M, Mathiesen C, Schaefer K, Thomsen KJ (2012). Neuronal inhibition and excitation, and the dichotomic control of brain hemodynamic and oxygen responses. NeuroImage.

[CR25] Lohani S, Poplawsky AJ, Kim SG, Moghaddam B (2016). Unexpected global impact of VTA dopamine neuron activation as measured by opto-fMRI. Mol Psychiatry.

[CR26] Lymperopoulos A, Bathgate A (2013). Arrestins in the cardiovascular system. Prog Mol Biol Transl Sci.

[CR27] Mandeville JB, Sander CY, Jenkins BG, Hooker JM, Catana C, Vanduffel W, Alpert NM, Rosen BR, Normandin MD (2013). A receptor-based model for dopamine-induced fMRI signal. NeuroImage.

[CR28] Mandeville JB, Liu CH, Vanduffel W, Marota JJ, Jenkins BG (2014). Data collection and analysis strategies for phMRI. Neuropharmacology.

[CR29] Masri B, Salahpour A, Didriksen M, Ghisi V, Beaulieu JM, Gainetdinov RR, Caron MG (2008). Antagonism of dopamine D2 receptor/beta-arrestin 2 interaction is a common property of clinically effective antipsychotics. Proc Natl Acad Sci U S A.

[CR30] Millan MJ, Maiofiss L, Cussac D, Audinot V, Boutin JA, Newman-Tancredi A (2002). Differential actions of antiparkinson agents at multiple classes of monoaminergic receptor. I. A multivariate analysis of the binding profiles of 14 drugs at 21 native and cloned human receptor subtypes. J Pharmacol Exp Ther.

[CR31] Nguyen TV, Brownell AL, Iris Chen YC, Livni E, Coyle JT, Rosen BR, Cavagna F, Jenkins BG (2000) Detection of the effects of dopamine receptor supersensitivity using pharmacological MRI and correlations with PET. Synapse 36 (1):57-65 10.1002/(SICI)1098-2396(200004)36:1<57::AID-SYN6>3.0.CO;2-K 10700026

[CR32] Olivera A, Eisner C, Kitamura Y, Dillahunt S, Allende L, Tuymetova G, Watford W, Meylan F, Diesner SC, Li L, Schnermann J, Proia RL, Rivera J (2010). Sphingosine kinase 1 and sphingosine-1-phosphate receptor 2 are vital to recovery from anaphylactic shock in mice. J Clin Invest.

[CR33] Park SM, Chen M, Schmerberg CM, Dulman RS, Rodriguiz RM, Caron MG, Jin J, Wetsel WC (2016). Effects of β-Arrestin-biased dopamine D2 receptor ligands on schizophrenia-like behavior in Hypoglutamatergic mice. Neuropsychopharmacology.

[CR34] Paxinos G, Franklin KBJ (2001). The mouse brain in stereotaxic coordinates.

[CR35] Peterson SM, Pack TF, Wilkins AD, Urs NM, Urban DJ, Bass CE, Lichtarge O, Caron MG (2015). Elucidation of G-protein and β-arrestin functional selectivity at the dopamine D2 receptor. Proc Natl Acad Sci U S A.

[CR36] Sander CY, Hooker JM, Catana C, Rosen BR, Mandeville JB (2016). Imaging agonist-induced D2/D3 receptor desensitization and internalization in vivo with PET/fMRI. Neuropsychopharmacology.

[CR37] Schwarz AJ, Danckaert A, Reese T, Gozzi A, Paxinos G, Watson C, Merlo-Pich EV, Bifone A (2006). A stereotaxic MRI template set for the rat brain with tissue class distribution maps and co-registered anatomical atlas: application to pharmacological MRI. NeuroImage.

[CR38] Shih YY, Chen CC, Shyu BC, Lin ZJ, Chiang YC, Jaw FS, Chen YY, Chang C (2009). A new scenario for negative functional magnetic resonance imaging signals: endogenous neurotransmission. J Neurosci.

[CR39] Shih YY, Chiang YC, Shyu BC, Jaw FS, Duong TQ, Chang C (2012). Endogenous opioid-dopamine neurotransmission underlie negative CBV fMRI signals. Exp Neurol.

[CR40] Skinbjerg M, Ariano MA, Thorsell A, Heilig M, Halldin C, Innis RB, Sibley DR (2009). Arrestin3 mediates D(2) dopamine receptor internalization. Synapse.

[CR41] Skinbjerg M, Liow JS, Seneca N, Hong J, Lu S, Thorsell A, Heilig M, Pike VW, Halldin C, Sibley DR, Innis RB (2010). D2 dopamine receptor internalization prolongs the decrease of radioligand binding after amphetamine: a PET study in a receptor internalization-deficient mouse model. NeuroImage.

[CR42] Urs NM, Gee SM, Pack TF, McCorvy JD, Evron T, Snyder JC, Yang X, Rodriguiz RM, Borrelli E, Wetsel WC, Jin J, Roth BL, O'Donnell P, Caron MG (2016). Distinct cortical and striatal actions of a β-arrestin-biased dopamine D2 receptor ligand reveal unique antipsychotic-like properties. Proc Natl Acad Sci U S A.

[CR43] Wang R, Foniok T, Wamsteeker JI, Qiao M, Tomanek B, Vivanco RA, Tuor UI (2006). Transient blood pressure changes affect the functional magnetic resonance imaging detection of cerebral activation. NeuroImage.

[CR44] Wehrl HF, Wiehr S, Divine MR, Gatidis S, Gullberg GT, Maier FC, Rolle AM, Schwenck J, Thaiss WM, Pichler BJ (2014). Preclinical and translational PET/MR imaging. J Nucl Med.

[CR45] Xu J, Hassanzadeh B, Chu W, Tu Z, Jones LA, Luedtke RR, Perlmutter JS, Mintun MA, Mach RH (2010). [3H]4-(dimethylamino)-N-(4-(4-(2-methoxyphenyl)piperazin-1-yl) butyl)benzamide: a selective radioligand for dopamine D(3) receptors. II. Quantitative analysis of dopamine D(3) and D(2) receptor density ratio in the caudate-putamen. Synapse.

