# Supplementary figures and images for: The role of beta-arrestin2 in shaping fMRI BOLD responses to dopaminergic stimulation
Source: Psychopharmacology (Berl). 2017 Apr 5;234(13):2019–30. doi: 10.1007/s00213-017-4609-6 (PMC5486931; doi:10.1007/s00213-017-4609-6)

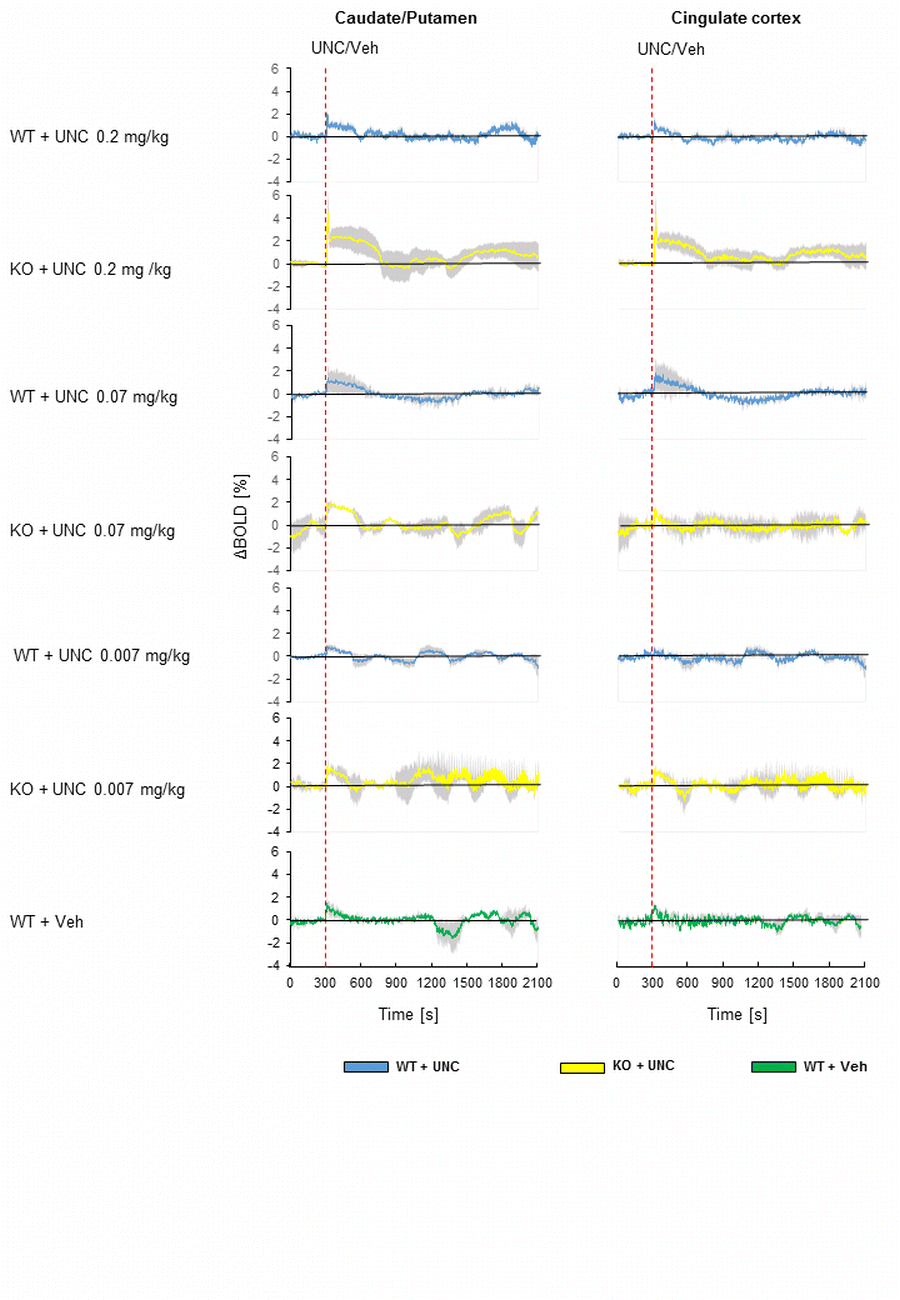

Supplement: Supplementary file 1 — BOLD signal responses in WT and beta-arrestin2 KO mice to UNC9994 (UNC), administered i.v. at doses of 0.2 (WT, n = 5; KO, n = 6), 0.07 (WT, n = 2; KO, n = 2), and 0.007 mg/kg (WT, n = 3; KO, n = 2), and to vehicle (Veh; WT, n = 2), in caudate/putamen and in cingulate cortex. Red dashed lines indicate the time of injection of UNC or vehicle. All data are shown as mean ± SEM (GIF 209 kb) [file 213_2017_4609_Fig7_ESM.gif]

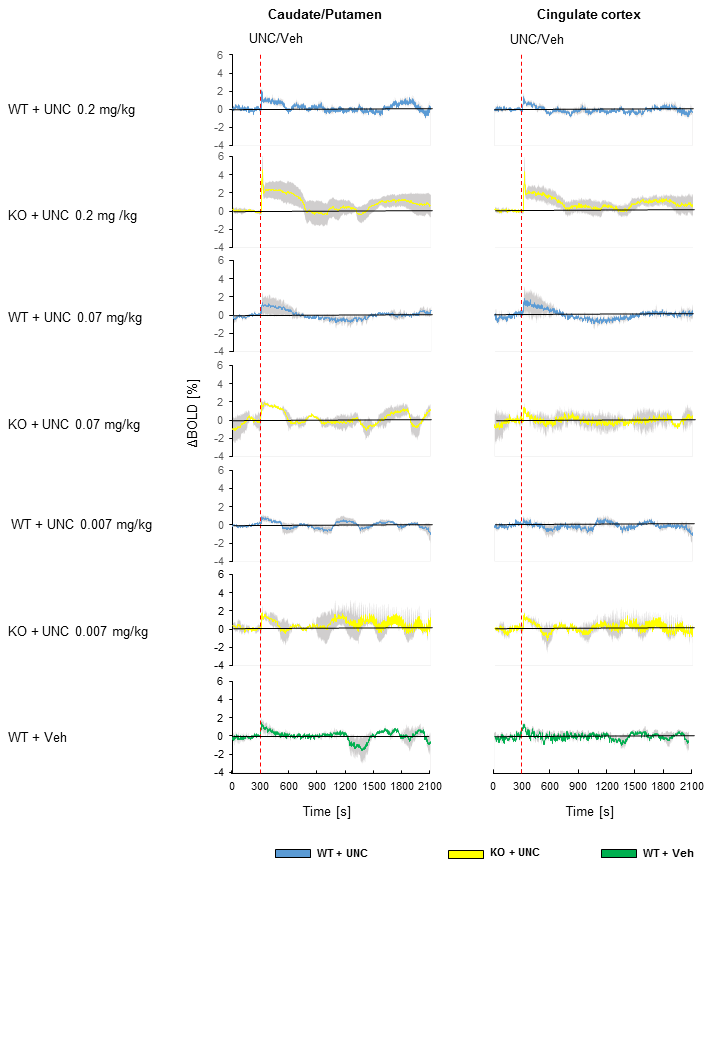

Supplement: Supplementary file 2 — High Resolution Image (TIFF 161 kb) [file 213_2017_4609_MOESM1_ESM.tif]
